# Dopamine D4 receptor activation restores CA1 LTP in hippocampal slices from aged mice

**DOI:** 10.1111/acel.12666

**Published:** 2017-10-03

**Authors:** Fangli Guo, Jianhua Zhao, Dandan Zhao, Jiangang Wang, Xiaofang Wang, Zhiwei Feng, Martin Vreugdenhil, Chengbiao Lu

**Affiliations:** ^1^ Key Lab of Brain Research of Henan Province Department of Physiology and Neurobiology Xinxiang Medical University Xinxiang China; ^2^ Department of Neurology 1st Affiliated Hospital of Xinxiang Medical University Xinxiang China; ^3^ Department of Psychology Xinxiang Medical University Xinxiang China; ^4^ Department of Health Sciences Birmingham City University Birmingham UK

**Keywords:** aging, dopamine, D4 receptor, LTP, memory

## Abstract

Normal aging is characterized with a decline in hippocampal memory functions that is associated with changes in long‐term potentiation (LTP) of the CA3‐to‐CA1 synapse. Age‐related deficit of the dopaminergic system may contribute to impairment of CA1 LTP. Here we assessed how the modulation of CA1 LTP by dopamine is affected by aging and how it is dependent on the Ca^2+^ source. In slices from adult mice, the initial slope of the field potential showed strong LTP, but in slices from aged mice LTP was impaired. Dopamine did not affect LTP in adult slices, but enhanced LTP in aged slices. The dopamine D1/D5 receptor (D1R/D5R) agonist SKF‐81297 did not affect LTP in adult but caused a relative small increase in LTP in aged slices; however, although there was no difference in dopamine D4 receptor (D4R) expression, the D4R agonist PD168077 increased LTP in aged slices to a magnitude similar to that in adult slices. The *N*‐Methyl‐D‐aspartate receptor antagonist D‐AP5 reduced LTP in adult slices, but not in aged slices. However, in the presence of D‐AP5, PD168077 completely blocked LTP in aged slices. The voltage‐dependent calcium channel (VDCC) blocker nifedipine reduced LTP in adult slices, but surprisingly enhanced LTP in aged slices. Furthermore, in the presence of nifedipine, PD168077 caused a strong enhancement of LTP in aged slices to a magnitude exceeding LTP in adult slices. Our results indicate that the full rescue of impaired LTP in aging by the selective D4R activation and that a large potentiation role on LTP by co‐application of D4R agonist and VDCC blocker may provide novel strategies for the intervention of cognitive decline of aging and age‐related diseases.

## Introduction

Normal brain aging is characterized by the decline in cognitive performance that includes modules dependent on hippocampal function, which has been linked to changes in long‐term potentiation (LTP) of synaptic strength in the hippocampus. (Shankar *et al*., 1998; Hsu *et al*., 2002; Rosenzweig & Barnes, 2003; Foster, 2007; Kumar, 2011). LTP of the Schaffer collateral (SC)‐CA1 synapse decreases with normal brain aging when using low‐intensity LTP induction protocols that mainly rely on Ca^2+^ influx through *N*‐Methyl‐D‐aspartate receptors (NMDARs) (Hsu *et al*., 2002), but not when high‐intensity protocols are used (Kumar, 2011). The aging‐dependent increase in expression and function of voltage‐dependent calcium channels (VDCCs) in CA1 pyramidal neurons is thought to cause inhibition of *N*‐Methyl‐D‐aspartate receptors (NMDARs) (Norris *et al*., 1996; Shankar *et al*., 1998). Consequently, aging is associated with a shift in synaptic plasticity from NMDAR‐dependent mechanisms to VDCC‐dependent mechanisms (Shankar *et al*., 1998; Foster, 2007; Kumar, 2011).

The aging‐related decline in episodic memory has also been associated with the age‐dependent decline in dopaminergic neurotransmission (Chowdhury *et al*., 2012), which is known to modulate hippocampal synaptic transmission, synaptic plasticity, and learning and memory (Lisman & Grace, 2005; Granado *et al*., 2008; Puig *et al*., 2014). The aging‐related changes in hippocampal dopamine receptor (DR) expression are, however, controversial. Whereas Hernandez *et al*. (2014) report an aging‐related increase in the expression of dopamine D2, D3, and D5 receptors, others found a decrease in the expression of dopamine receptors in the hippocampus from humans and rodents (Amenta *et al*., 2001; Hemby *et al*., 2003; Backman *et al*., 2006).

In the adult hippocampus, activation of the D_1_‐type dopamine D1 receptor (D1R) increases the early LTP magnitude at CA1 hippocampal synapses (Otmakhova & Lisman, 1996) and stabilizes protein synthesis‐dependent late LTP both *in vitro* (Huang & Kandel, 1995) and *in vivo* (Lemon & Manahan‐Vaughan, 2006). Remarkably, although D_2_‐type DRs have opposite cellular effects, D2 receptor and D3 receptor activation are also involved in the induction and maintenance of LTP in the CA3‐CA1 synapse (Swant & Wagner, 2006).

The dopamine D4 receptor (D4R) is the most abundant D_2_‐type DR in the hippocampus. D4R activation reduced LTP in stratum oriens, but not in stratum radiatum (SR) of the hippocampus (Herwerth *et al*., 2012; Li *et al*., 2016). However, Kwon *et al*. (2008) showed that D4R activation de‐potentiates LTP in SR by reducing AMPA receptor surface expression and currents.

Interestingly, in the prefrontal cortex D4R seems to serve as a synaptic stabilizer, maintaining synaptic homeostasis, as it positively or negatively modulates AMPAR‐mediated synaptic current, depending on the neuronal excitability level. Whereas D4R activation causes up‐regulation of AMPAR function when neurons are in a state of low excitability (Yuen *et al*., 2010), it reduces excitatory transmission in a stress‐related state of enhanced neuronal excitability (Yuen *et al*., 2013). In normal aging, the intrinsic excitability of CA1 pyramidal neurons is reduced, due to an enhanced slow afterhyperpolarization caused by increased VDCC activity (Tombaugh *et al*., 2005). It is therefore possible that in the aged hippocampus D4R activation may upregulate synaptic potentiation in an attempt to restore excitability by alteration of intracellular Ca^2+^ homeostasis. To test whether aging affects the dopaminergic modulation of synaptic plasticity in a Ca^2+^ influx‐dependent way, we examined the effect of selective DR agonists on hippocampal CA1 LTP in the presence of NMDA receptor antagonist or VDCC blocker. We report here that aging‐related impairment of CA1 LTP can be fully reversed by D4R activation, most potently in the presence of a VDCC blocker.

## Results

### Aging reduces LTP of the Schaffer collateral‐CA1 synapse

**Figure 1:**
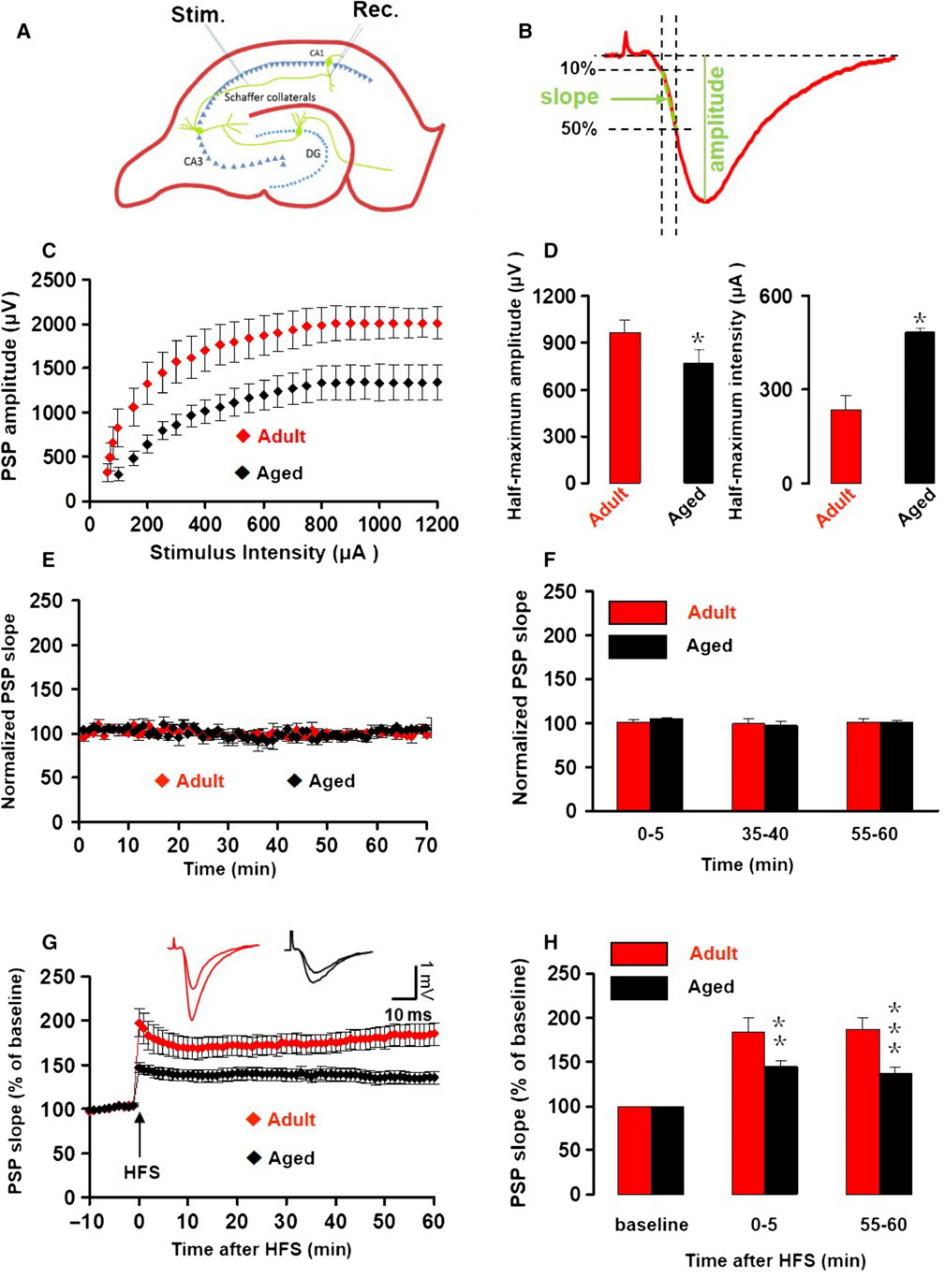
Aging‐dependent decline of LTP. (A) Location of stimulus electrode and recording electrode in mouse ventral hippocampal slices. (B) CA1 population synaptic potential (PSP) measurements. The PSP amplitude was taken as difference between the baseline and the PSP trough. The downward slope was taken from 10% to 50% of the PSP amplitude. (C) Stimulus–response relationship. CA1 PSP amplitude as function of SC stimulus intensity for 7 slices from adult mice (red diamonds) and 11 slices from aged mice (black diamonds). Data give the mean ± SEM. (D) The half‐maximum amplitude of the PSP and the stimulus intensity required for half‐maximum PSP amplitude for slices from adult mice (red bars) and aged mice (black bars). Data give the mean and SEM. Student's *t*‐test probability for comparisons between slices from adult and aged is given as *, *P* < 0.05. (E) The PSP slope normalized to the average of the PSP slope over the first 10 min (baseline) for six slices from adult mice and five slices from aged mice. Insets give representative traces illustrating the average PSP over the first 5 min and after 55–60 min. Details as in C. (F) Histogram gives the PSP slope as percentage of the first 10 min for different epochs, for slices from adult mice (red bars) and aged mice (black bars). Details as in D. (G) The PSP slope normalized to the average of the PSP slope over 10‐min pre‐HFS baseline, for 12 slices from adult mice and 11 slices from aged mice. Details as in C. Insets give representative traces illustrating the average PSP over the 5 min prior to HFS and 25–30 min post‐HFS. The left pair of traces is from an adult mouse; the right pair is from an aged mouse. HFS: high‐frequency stimulation. (H) Histogram gives the HFS‐induced LTP magnitude (percentage increase in PSP slope relative to baseline) for immediate LTP (average over 0–5 min post‐HFS) and early LTP (average over 55–60 min post‐HFS), for slices from adult mice (red bars) and aged mice (black bars). Data give the mean and SEM. Student's *t*‐test was used for comparisons between slices from adult and aged mice: **, *P* < 0.01; ***, *P* < 0.001.

Aging has been associated with reduced LTP of the SC‐CA1 synapse. To test this, the population synaptic potential (PSP) was recorded in SR of the CA1 area in response SC stimulation (Fig. 1A). To determine the standard stimulation intensity for each slice, stimuli were given at varying stimulus intensity and the PSP amplitude (example in Fig. 1B) was used to construct a stimulus–response curve. This was used to determine the half‐maximum amplitude and the standard stimulus intensity that elicited the half‐maximum PSP amplitude. Compared to that in adult controls (3–4 months), the PSP evoked in slices from aged mice (20–24 months) had a reduced maximum amplitude (*T*_16_ = 2.82, *P* = 0.012. Figure 1C,D), whereas the half‐maximum PSP amplitude was achieved at a higher stimulus intensity (*T*_16_ = 2.473, *P *= 0.025. Figure 1D). This half‐maximum stimulus intensity was then used for both test stimuli and LTP induction. To quantify changes in the SC‐CA1 synapse, we used the early PSP slope (example in Fig. 1B). The early slope is likely to reflect mainly excitatory synaptic currents and is unlikely to be contaminated by the population spike. We tested the stability of the PSP slope as function of time in control artificial cerebrospinal fluid (aCSF) after a 10‐min stabilizing period. Both in slices from adult and aged mice, the PSP slope was stable for over 1 h (Fig. 1E). Compared to the PSP slope at 0–5 min, the percentage change of PSP slope at 55–60 min was 0.22 ± 4.72% (*F*_4_ = 0.016, *P* = 0.984) and −1.35 ± 4.68% (*F*_3_ = 1.188, *P* = 0.368) for adult and aged slices, respectively. Repeated‐measures ANOVA showed no difference between the PSP slope development over 1 h in aged slices compared to adult slices *F*_2,12_ = 0.658, *P* = 0.536 (Fig. 1F).

A single high‐frequency stimulus (HFS) train (100 Hz, 1s) was used to evoke LTP.

We quantified the HFS‐induced SC‐CA1 LTP by normalizing the PSP slope to the pre‐HFS baseline over the first 5 min post‐HFS: immediate phase of LTP (iLTP) and over 55–60 min after HFS: early LTP (eLTP). Both iLTP and eLTP were smaller in slices from aged mice than that in slices from adult controls (Fig. 1G,H); iLTP was 46.1% smaller (*T*_21_ = 3.359, *P* = 0.003, Fig. 1F) in slices from aged mice (45.3 ± 6.0% of baseline), compared to that in adult controls (84.0 ± 15.6% of baseline). eLTP was 57.6% smaller in aged slices (adult: 86.8 ± 12.3% vs. aged: 36.8 ± 7.1%; *T*_21_ = 4.521, *P* < 0.001, Fig. 1F). These results confirm that LTP of the SC‐CA1 synapse is impaired in aged mice.

### Dopamine reverses the aging‐associated impairment of CA1 LTP through the D4 receptor in slices from aged mice

**Figure 2:**
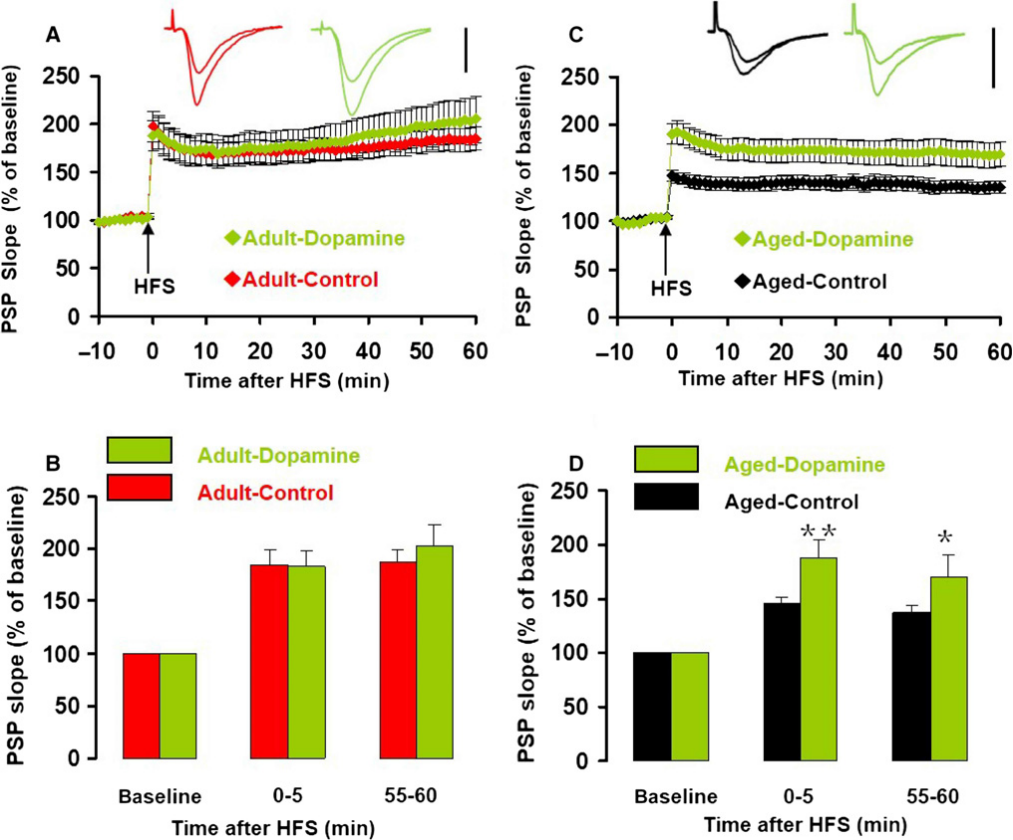
Dopamine had no effect on LTP in adult mice, but increased LTP in aged mice. (A) The normalized PSP slope recorded in slices from adult mice, in the presence (green) and absence (red) of dopamine (200 μm). Insets give representative traces illustrating the increase in PSP slope. Data give the mean ± SEM. (B) Histogram of the normalized PSP for baseline, immediate LTP, and early LTP. Data give the mean ± SEM for recordings in vehicle control (*n* = 12, red bars) and dopamine (*n* = 8, green bars). (C) The normalized PSP slope recorded in slices from aged mice, in the presence (green) and absence (black) of dopamine. Details as in A. (D) Histogram of the normalized PSP for baseline, iLTP, and eLTP in vehicle control (*n* = 11, black bars) and dopamine (*n* = 8, green bars). Data give the mean and SEM. Levels of significance for post hoc paired *t*‐test after two‐way ANOVA for repeated measurements: *P < 0.05; **P < 0.01.

DR activation modulates LTP of the SC‐CA1 synapse (Navakkode *et al*., 2012). To determine whether the effects of DR activation can restore the aging‐associated impairment of CA1 LTP, we tested the effect of the natural DR ligand dopamine, the selective D1R/D5R agonist SKF‐81297, and the D4R agonist PD168077 on the HFS‐induced LTP in hippocampal slices from both adult and aged mice. Application of dopamine (200 μm) had no effect on the half‐maximum amplitude in slices from adult mice (*T*_13_ = 0.745, *P* = 0.470, compared to control) and aged mice (*T*_17_ = 0.745, *P* = 0.394), or on the half‐maximum intensity in slices from adult mice (*T*_15_ = 0.246, *P* = 0.809, compared to control) and aged mice (*T*_17_ = 0.457, *P* = 0.653). Dopamine had no effect on either iLTP or eLTP in adult mice (two‐way ANOVA; main effect of drug, *F*_1,18_ = 0.000, *P* = 0.987, main effect of time, *F*_1,18_ = 2.125, *P* = 0.162, Fig. 2A,B), but caused a significant increase in LTP in slices from aged mice (two‐way ANOVA; main effect of drug, *F*_1,17_ = 6.939, *P* = 0.017, main effect of time, *F*_1,17_ = 19.138, *P* < 0.001). Dopamine increased iLTP by 93.6% (dopamine: 87.8 ± 11.5% vs. control: 45.3 ± 6.0%; *T*_17_ = 0.745, *P* = 0.002, Fig. 2C,D) and eLTP by 88.5% (dopamine: 69.3 ± 14.0% vs. control: 36.8 ± 7.1%, *T*_17_ = 0.046, *P* = 0.046, Fig. 2C,D). In the presence of dopamine, there was no difference in LTP (*F*_1,14_ = 0.344, *P* = 0.567, Fig. 2B,D) between slices from adult and aged mice.

**Figure 3:**
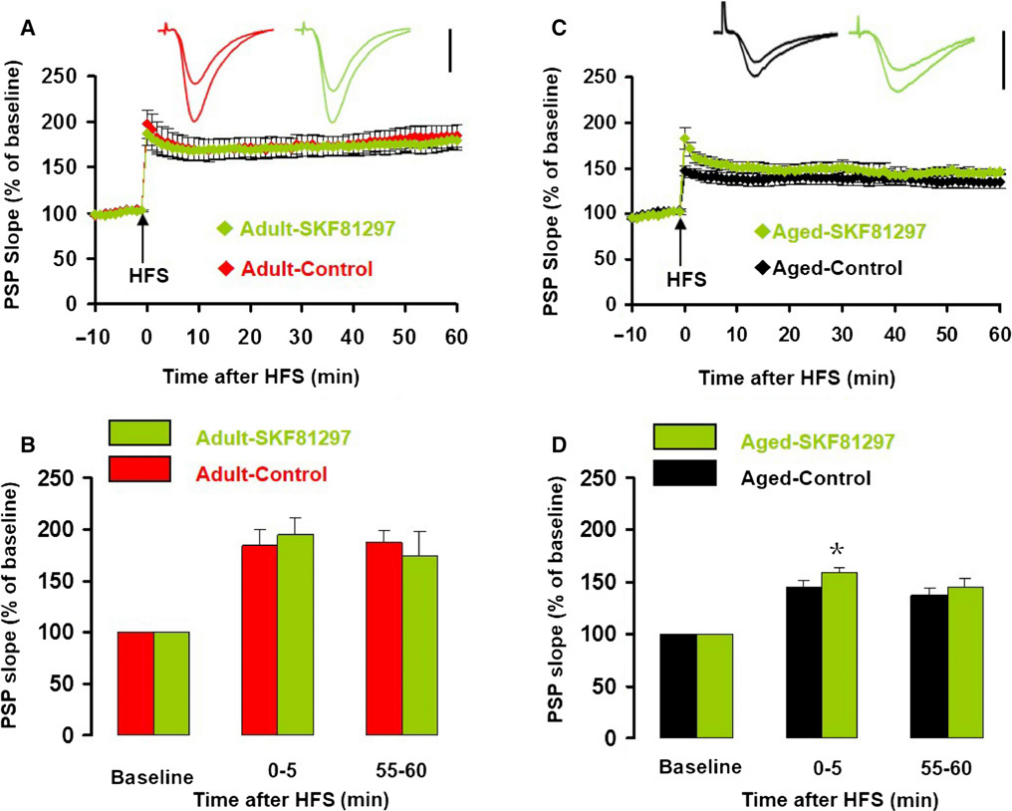
The D1R agonist SKF‐81297 had no effect on LTP. (A) The normalized PSP slope recorded in slices from adult mice, in the presence (green) and absence (red) of SKF‐81297 (1 μm). Insets give representative traces illustrating the increase in PSP slope. Data give the mean ± SEM. (B) Histogram of the normalized PSP for baseline, iLTP, and eLTP. Data give the mean and SEM for recordings in vehicle control (*n* = 12, red bars) and SKF‐81297 (*n* = 8, green bars). (C) The normalized PSP slope recorded in slices from aged mice, in the presence (green) and absence (black) of SKF‐81297. Details as in A. (D) Histogram of the normalized PSP for baseline, iLTP, and eLTP in vehicle control (*n* = 11, black bars) and SKF‐81297 (*n* = 8, green bars). Data give the mean and SEM. Level of significance for post hoc paired *t*‐test after two‐way ANOVA for repeated measurements: *P < 0.05.

To determine which DR type is responsible for the dopamine‐mediated reversal of age‐related decline in CA1 LTP, we tested the effect of DR type selective agonists. Application of the D1R/D5R agonist SKF‐81297 (1 μm) had no effect on the half‐maximum amplitude in slices from adult mice (*T*_12_ = 1.56, *P* = 0.145, vs. control) and aged mice (*T*_20_ = 0.472, *P* = 0.394) or half‐maximum intensity in slices from adult mice (*T*_14_ = 1.77, *P* = 0.108, vs. control) and aged mice (*T*_20_ = 0.991, *P* = 0.334). Application of SKF‐81297 had no effect on the magnitude of iLTP or eLTP (*F*_1,18_ = 0.760, *P* = 0.395, Fig. 3A,B). In slices from aged mice, SKF‐81297 caused a small increase in iLTP (by 28.2%, SKF‐81297: 58.1 ± 5.0% vs. control: 45.3 ± 6.0%; *T*_17_ = 2.272, *P* = 0.036) but not in eLTP (*T*_17_ = 0.956, *P* = 0.352, Fig. 3C,D).

**Figure 4:**
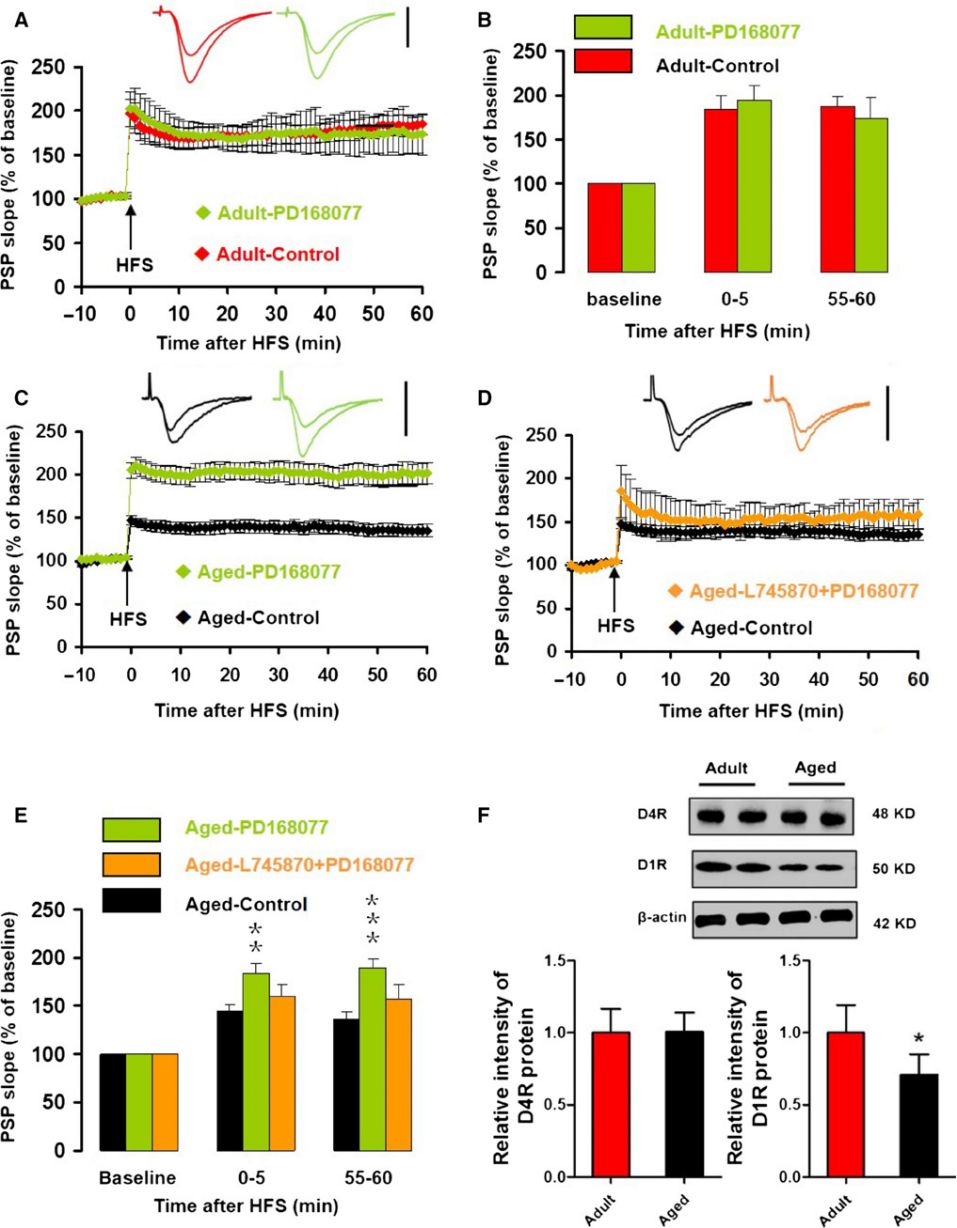
The D4R agonist PD168077 had no effect on LTP in adult mice but enhanced LTP in aged mice. (A) The normalized PSP slope recorded in slices from adult mice, in the presence (green) and absence (red) of PD168077 (200 nm). Insets give representative traces illustrating the increase in PSP slope. Data give the mean ± SEM (B) Histogram of the normalized PSP for baseline, iLTP, and eLTP. Data give the mean and SEM for recordings in vehicle control (*n* = 12, red bars) and PD168077 (*n* = 9, green bars). (C) The normalized PSP slope recorded in slices from aged mice, in the presence (green) and absence (black) of PD168077. Details as in A. (D) The normalized PSP slope recorded in slices from aged mice, in the presence (orange) and absence (black) of PD168077 (200 nm) plus the selective D4R antagonist L745870 (200 nm). Details as in A. (E) Histogram of the normalized PSP for baseline, iLTP, and eLTP in vehicle control (*n* = 11, black bars), PD168077 (*n* = 8, green bars), and PD168077 + L745870 (*n* = 4, orange bars). Data give the mean and SEM. Level of significance for post hoc paired *t*‐test after two‐way ANOVA for repeated measurements: **P < 0.01; ***P < 0.001. (F) Aging‐related changes of DR expression in the mouse hippocampus. Expression of D4R, D1R, and β‐actin was assessed by Western blotting (inset). Histograms give the expression of D4R protein (left panel) and D1R protein (right panel) normalized to the expression of β‐actin in the hippocampus of six adult mice (red bars) and six aged mice (black bars). Data give the mean and SEM. * indicates Student's paired *t*‐test *P < *0.05.

Application of the D4R agonist, PD168077 (200 nm) had no effect on the half‐maximum amplitude (two‐way ANOVA; main effect of drug, *F*_1,36_ = 1.370, *P* = 0.249, main effect of age, *F*_1,36_ = 17.588, *P < *0.001, Fig. 4B,E) and the half‐maximum intensity (two‐way ANOVA; main effect of age, *F*_1,39_ = 5.619, *P* = 0.023, main effect of drug, *F*_1,36_ = 0.062, *P* = 0.805) in slices from adult mice and aged mice. In slices from adult mice, PD168077 had no effect on iLTP or eLTP (two‐way ANOVA; main effect of time, *F*_1,19_ = 0.166, *P* = 0.689, main effect of drug, *F*_1,19_ = 0.909, *P* = 0.352, Fig. 4A,B). However, in aged slices, PD168077 increased iLTP by 82.9% (PD168077: 82.9 ± 11.0% vs. control: 45.3 ± 6.0%; *T*_17_ = 3.609, *P* = 0.002, Fig. 4C,E) and eLTP by 142.4% (PD168077: 89.2 ± 10.1% vs. control: 36.8 ± 7.1%; *T*_17_ = 4.293, *P < *0.001, Fig. 4C,E).

In the presence of PD168077, there was no difference in LTP magnitude between slices from adult and aged mice (two‐way ANOVA; main effect of drug, *F*_1,37_ = 3.444, *P* = 0.071, main effect of age, *F*_1,37_ = 4.013, *P* = 0.053, Fig. 4B,E). To confirm that the effect of PD168077 is mediated by D4R activation, we repeated the experiment in the presence of the highly selective D4R antagonist L745870. In slices from aged mice, L745870 (200 nm) completely prevented the effect of PD168077 on iLTP (*T*_10_ = 2.497, *P* = 0.032) and eLTP (*T*_10_ = 2.332, *P* = 0.042, Fig. 4D). These results revealed that, whereas DR activation did not affect CA1 LTP in slices from adult mice, dopamine, through D4R activation, restored LTP in slices from aged mice to the same magnitude as that in adult controls.

### Aging‐related changes in D4R and D1R expression

To determine whether the age‐dependent effects of D4R activation on CA1 LTP is due to differential levels of DR subtype expression, we quantified the expression of D1R and D4R in hippocampal tissue from adult and aged mice through Western blots. D1R or D4R expression was normalized to the expression of β‐actin in the same tissue (example in inset of Fig. 4F). D1R expression was reduced by 29% with aging (*T*_5_ = 2.579, *P* = 0.049, Fig. 4F), but D4R expression levels were not different with aging (*T*_11_ = 0.046, *P* = 0.964, Fig. 4F). This indicates that the increased effect of D4R receptor activation on CA1 LTP is unlikely the result of an increased D4R expression in the hippocampus.

### The effects of NMDA receptor and voltage‐dependent calcium channel blockers on LTP in adult and aged mice

**Figure 5:**
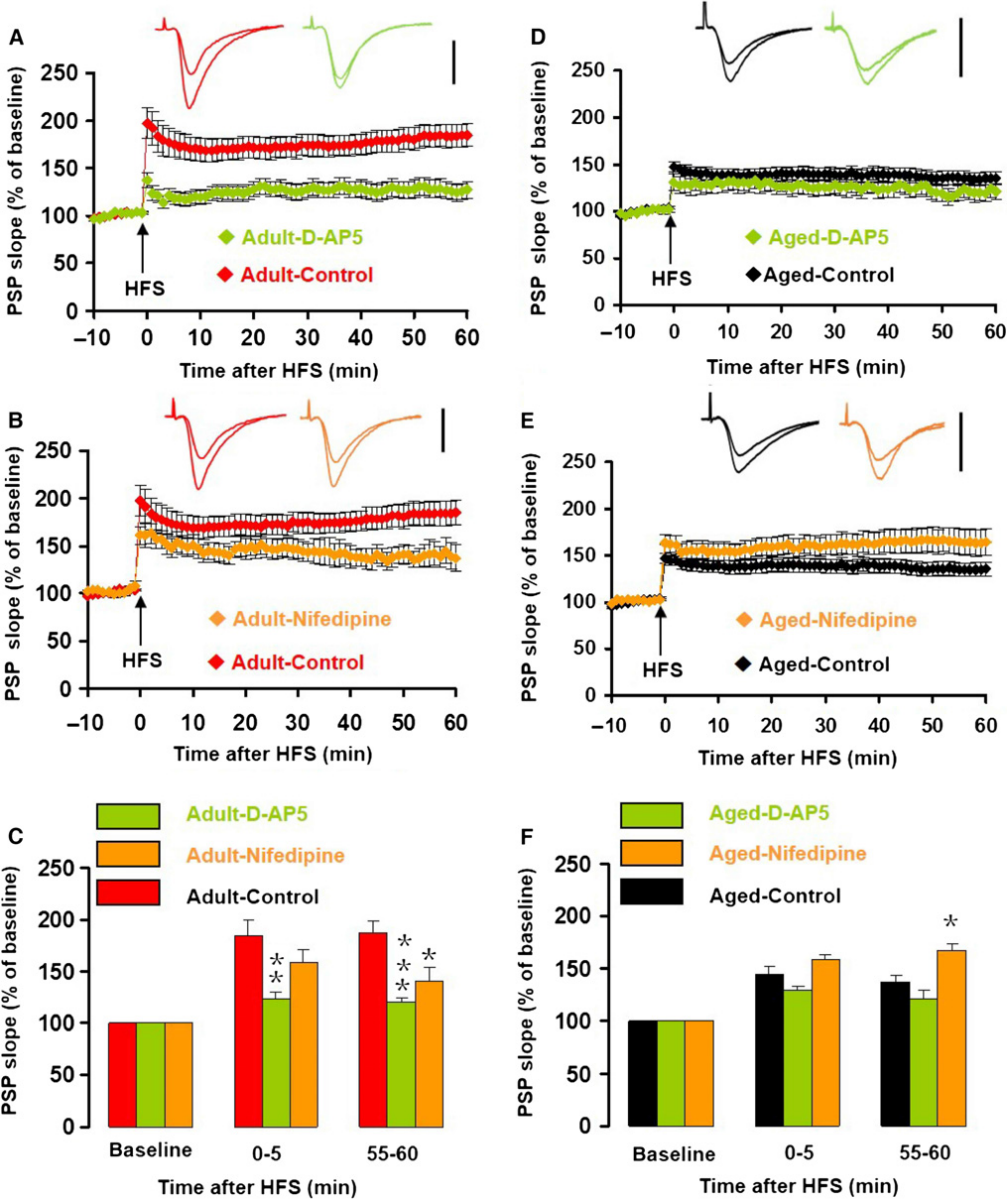
Age‐related changes in NMDAR‐ or VDCC‐dependent LTP. (A) The normalized PSP slope recorded in slices from adult mice, in the presence (green) and absence (red) of the NMDAR antagonist D‐AP5 (50 μm). Insets give representative traces illustrating the increase in PSP slope. Data give the mean ± SEM. (B) The normalized PSP slope recorded in slices from adult mice, in the presence (orange) and absence (red) of the VDCC blocker nifedipine (30 μm). Details as in A. (C) Histogram of the normalized PSP for baseline, iLTP, and eLTP. Data give the mean and SEM for recordings in vehicle control (*n* = 12, red bars), D‐AP5 (*n* = 6, green bars), and nifedipine (*n* = 4, orange bar). Level of significance for post hoc paired *t*‐test after two‐way ANOVA for repeated measurements: **P < 0.01; ***P < 0.001. (D) The normalized PSP slope recorded in slices from aged mice, in the presence (green) and absence (black) of D‐AP5. Details as in A. (E) The normalized PSP slope recorded in slices from aged mice, in the presence (orange) and absence (black) of nifedipine. Details as in A. (F) Histogram of the normalized PSP for baseline, iLTP, and eLTP in vehicle control (*n* = 11, black bars), D‐AP5 (*n* = 5, green bars), and nifedipine (*n* = 7, orange bar). Level of significance for post‐hoc paired *t*‐test after two‐way ANOVA for repeated measurements: *P < 0.05.

With aging, the dependence of CA1 LTP on Ca^2+^ influx through NMDARs reduces and the dependence on Ca^2+^ influx through VDCCs increases (Shankar *et al*., 1998). To test whether similar changes could play a role in the age‐dependent effect of dopamine on LTP, we tested the effect of the NMDAR antagonist D‐AP5 and the VDCC blocker nifedipine on LTP. Application of D‐AP5 (50 μm) had no effect on the half‐maximum amplitude in slices from adult mice (*T*_8_ = 0.701, *P* = 0.503, vs. control) and aged mice (*T*_8_ = 0.281, *P* = 0.786) or half‐maximum intensity in slices from adult mice (*T*_11_  = 2.089, *P* = 0.06, vs. control) and aged mice (*T*_8_ = 2.012, *P* = 0.079). Application of nifedipine (30 μm) had no effect on the half‐maximum amplitude in slices from adult mice (*T*_9_ = 0.981, *P* = 0.352, vs. control) and aged mice (*T*_10_ = 1.014, *P* = 0.335) or half‐maximum intensity in slices from adult mice (*T*_11_ = 0.129, *P* = 0.900, vs. control) and aged mice (*T*_10_ = 1.737, *P* = 0.113). This suggests that NMDARs and VDCCs have no significant contribution to SC‐CA1 synapse at baseline conditions. In slices from adult mice, D‐AP5 caused a 73.1% decrease in iLTP (D‐AP5: 22.6 ± 7.1% vs. control: 84.0 ± 15.6%; *T*_16_ = 3.37, *P* = 0.004, Fig. 5A,C) and a 76.8% decrease in eLTP (D‐AP5: 20.1 ± 3.9% vs. control: 86.8 ± 12.3%; *T*_16_ = 4.836, *P < *0.001, Fig. 5A,C). However, in slices from aged mice, D‐AP5 had no effect on iLTP (*T*_14_ = 0.752, *P* = 0.465) or eLTP (*T*_14_ = 0.513, *P* = 0.616, Fig. 5D,F). In the presence of D‐AP5, there was no difference in iLTP or eLTP between slices from adult and aged mice (two‐way ANOVA; main effect of age, *F*_1,9_ = 0.796, *P* = 0.396, main effect of time, *F*_1,9_ = 0.802, *P* = 0.394, Fig. 5C,F).

When applied to slices from adult mice, nifedipine (30 μm) had no effect on iLTP (*T*_14_ = 1.263, *P* = 0.227), but reduced eLTP by 53.3% (nifedipine: 40.5 ± 13.3% vs. control: 86.8 ± 12.3; *T*_14_ = 2.485, *P* = 0.027, Fig. 5B,C). In contrast, in slices from aged mice, nifedipine increased eLTP by 84.4% (nifedipine: 67.8 ± 15.7% vs. control: 36.8 ± 7.1%; *T*_16_ = 2.525, *P* = 0.023, Fig. 5E,F), but had no significant effect on iLTP (*T*_16_ = 1.212, *P* = 0.243). In the presence of nifedipine, there was no difference in iLTP or eLTP between slices from adult and aged mice (two‐way ANOVA; main effect of age, *F*_1,9_ = 0.270, *P* = 0.616, main effect of time, *F*_1,9_ = 0.368, *P* = 0.559), Fig. 5C,F).

These results indicate that CA1 LTP in adult mice is dependent on Ca^2+^ influx through both NMDARs and VDCCs. The small CA1 LTP remaining in aged mice was not dependent on NMDAR activation and Ca^2+^ influx through VDCCs seems even to suppress LTP.

### D4R‐mediated increase in LTP in aged mice was potentiated by nifedipine

To understand the role of D4R activation on LTP in slices from aged mice, we pretreated hippocampal slices with the NMDAR antagonist D‐AP5 or the VDCC blocker nifedipine for 20 min, followed by an application of the D4R agonist PD168077 for 20 min, and then applied HFS to induce LTP.

**Figure 6:**
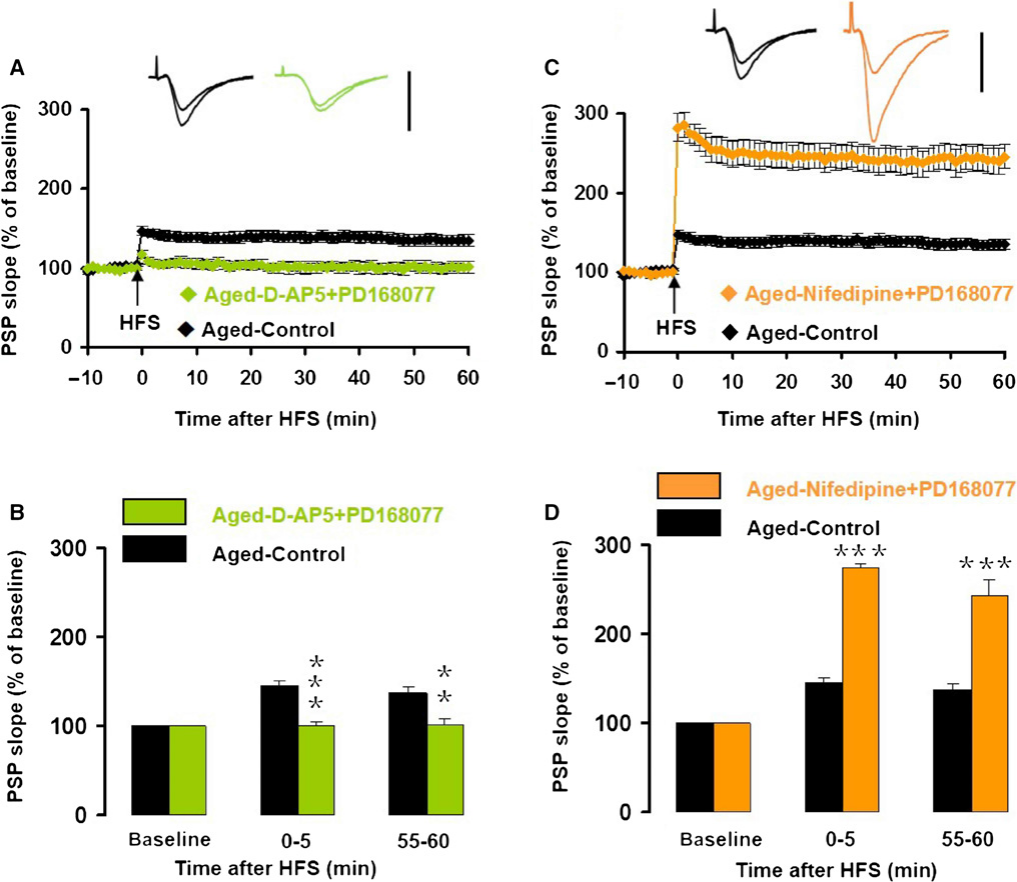
The D4R agonist PD168077‐induced increase of LTP in slices from aged mice was potentiated by the VDCC blocker nifedipine, but suppressed by the NMDAR antagonist D‐AP5. (A) The normalized PSP slope recorded in slices from aged mice, in the presence (green) and absence (black) of D‐AP5 (50 μm) plus PD168077 (200 nm). Insets give representative traces illustrating the increase in PSP slope. Data give the mean ± SEM. (B) Histogram of the normalized PSP for baseline, iLTP and eLTP. Data give the mean and SEM for recordings in vehicle control (*n* = 11, black bars) and D‐AP5 plus PD168077 (*n* = 7, green bars). Level of significance for post hoc paired *t*‐test after two‐way ANOVA for repeated measurements: **P < 0.01; ***P < 0.001. (C) The normalized PSP slope recorded in slices from aged mice, in the presence (orange) and absence (black) of nifedipine (530 μm) plus PD168077 (200 nm). Details as for A. (D) Histogram of the normalized PSP for baseline, iLTP, and eLTP of recordings in vehicle control (*n* = 12, black bars) and nifedipine plus PD168077 (*n* = 7, green bars). Details as in B.

Application of D‐APV + PD168077 had no effect on the half‐maximum amplitude *T*_20_ = 0.788, *P* = 0.439, vs. control) or half‐maximum intensity (*T*_20_ = 1.517, *P* = 0.145, vs. control) in aged mice. Application of nifedipine + PD168077 had no effect on the half‐maximum amplitude (*T*_16_ = 0.473, *P* = 0.641, vs. control) or half‐maximum intensity (*T*_16_ = 1.083, *P* = 0.295, vs. control). In the presence of D‐AP5 and PD168077, HFS failed to induce either iLTP (99 ± 5% of baseline, *T*_16_ = 4.380, *P < *0.001, compared to control) or eLTP (101 ± 6% of baseline, *T*_16_ = 3.182, *P* = 0.006, compared to control, Fig. 6A,B). However, in the presence of nifedipine, PD168077 increased iLTP by 283.6% (nifedipine + PD168077: 174.0 ± 5.6% vs. control: 45.3 ± 6.0%, *T*_15_ = 6.958, *P < *0.001; and *T*_12_ = 2.994, *P* = 0.011, compared to PD168077 only) and increased eLTP by 288.4% (nifedipine + PD168077: 142.8 ± 18.0% vs. control: 36.8 ± 7.1%; *T*_15_ = 4.955, *P < *0.001, and *T*_12_ = 1.583, *P* = 0.139, compared to PD168077 only, Fig. 6C,D). The D4R‐induced increase in LTP magnitude in the presence of nifedipine was more than the effect of nifedipine alone (84%) plus the effect of D4R alone (142%). These results indicate that the D4R activation‐induced increase in CA1 LTP in aged mice requires Ca^2+^ influx through NMDARs, whereas Ca^2+^ influx through VDCCs seems to suppress CA1 LTP.

## Discussion

In this study, we report that normal brain aging is associated with an impairment of HFS‐induced SC‐CA1 LTP and that D4R activation of restored LTP in aged mice to levels found in adult mice. Furthermore, we observed that the LTP‐enhancing effect of D4R activation was potentiated by blocking VDCCs, whereas D4R activation potentiated the LTP suppression caused by NMDAR inhibition.

### Aging impairs SC‐CA1 LTP

The SC‐CA1 synapse can express NMDAR‐dependent LTP and VDCC‐dependent LTP (Kumar, 2011). The Ca^2+^ influx through NMDAR activates calcium–calmodulin‐dependent protein serine/threonine kinase II that increases AMPA receptor conductance and insertion of additional AMPA receptors in the activated postsynaptic density (Lisman, 2014). The reduction in SC‐CA1 synapse LTP with normal brain aging confirmed previous observations (Hsu *et al*., 2002). However, others report no difference in LTP magnitude, but claim that aging causes a shift in synaptic plasticity from NMDAR‐dependent mechanisms to VDCC‐dependent mechanisms (Shankar *et al*., 1998; Kumar, 2011). This may be because with aging Ca^2+^ influx through NMDARs is reduced (Bodhinathan *et al*., 2010) and Ca^2+^ influx through L‐type VDCCs is increased (Norris *et al*., 1996; Shankar *et al*., 1998). The effect of aging on CA1 LTP is therefore dependent on the LTP induction method that determines what source of Ca^2+^ influx is involved. When using strong LTP induction protocols, like four HFS trains, aged rats showed no change in CA1 LTP (Shankar *et al*., 1998), whereas theta‐induced, NMDA‐dependent LTP was reduced (Tombaugh *et al*., 2002).

Under our single HFS train LTP induction protocol, CA1 LTP in adult mice was partly NMDAR‐dependent and partly VDCC‐dependent. The LTP remaining in slices from aged mice was not affected by the NMDAR antagonist D‐AP5, which may be explained by an aging‐associated limit in postsynaptic depolarization, essential for NMDA‐dependent LTP (Kumar, 2011). In contrast to other studies who report that LTP in the aged CA1 is more VDCC‐dependent (Shankar *et al*., 1998; Kumar, 2011), in our hands blocking VDCCs with nifedipine did not suppress LTP in slices from aged mice, but even enhanced it. Under the assumption that L‐type VDCC activity in mouse CA1 pyramidal cells would be increased in aging, like in rats (Shankar *et al*., 1998), it is counterintuitive that reducing VDCC‐dependent Ca^2+^ influx would facilitate LTP. It may be explained by a decrease in NMDAR activation, because NMDARs are inactivated by high intracellular Ca^2+^ concentration (Rosenmund *et al*., 1995) and elevated intracellular Ca^2+^ concentration increases the threshold frequency for induction of LTP (Shankar *et al*., 1998; Ris & Godaux, 2007). Alternatively, blocking VDCCs may increase CA1 pyramidal cell excitability, because it will reduce the slow afterhyperpolarization, which is increased in CA1 neurons, due to the increase in VDCCs (Tombaugh *et al*., 2005).

Whether aging reduces NMDAR‐dependent LTP or replaces it with VDCC‐dependent LTP (Kumar, 2011), the changes in LTP will, together with increased tendency for synaptic depression (Rosenzweig & Barnes, 2003), contribute to the impaired memory functions observed in normal aging (McNab *et al*., 2015). It is therefore important to explore ways to restore LTP to levels observed in adult mice.

### Dopamine does not affect CA1 LTP in adult mice

In our experiments, dopamine did not affect SC‐CA1 LTP in slices from adult mice, which is in line with other studies using HFS in SR (Herwerth *et al*., 2012). Selective activation of D_1_‐type DR had little effect on LTP, not dissimilar to the very small D_1_‐type DR‐mediated enhancement HFS‐induced CA1 LTP observed in rats (Otmakhova & Lisman, 1996). The protein synthesis‐dependent late CA1 LTP induced by repeated HFS trains was enhanced by D_1_‐type DR agonists (Huang & Kandel, 1995) and was reduced by D1R antagonists and in D1R knockout mice *in vitro* and *in vivo* (Granado *et al*., 2008; Ortiz *et al*., 2010), indicating that D1R activation is involved in the maintenance of CA1 LTP. Selective D_2_‐type D4R activation had little effect on LTP in slices from adult mice, which is in line with the finding of Herwerth *et al*. (2012), who showed that D4R activation had no effect on CA1 LTP using HFS in SR.

### D4R activation rescues LTP in slices from aged mice

In slices from aged mice, dopamine increased SC‐CA1 LTP to a magnitude similar to that in adult slices. This dopamine‐induced increase in LTP could be mimicked by selective D4R activation, but not by selective activation of D_1_‐type DRs. How D4R activation mediates the increase in SC‐CA1 LTP only in aged mice is currently not clear. It is unlikely caused by an age‐dependent increase in D4R expression because the Western blot analysis showed no difference with aging.

D4R activation causes hypofunction of NMDARs (Wang *et al*., 2003; Herwerth *et al*., 2012), which cannot explain the D4R‐mediated enhancement of LTP in aged mice. Interestingly, D4R activation can reduce VDCC‐mediated Ca^2+^ currents in prefrontal cortex neurons (Wang *et al*., 2006) and cerebellar granule cells via a G‐protein mediated mechanism. Because high intracellular Ca^2+^ concentration can inactivate NMDARs (Rosenmund *et al*., 1995) and increase the threshold frequency for induction of LTP (Shankar *et al*., 1998; Ris & Godaux, 2007), inhibition of VDCCs in aged cells may cause a recovery of NMDA‐mediated LTP. This is confirmed by the observation that in the presence of D‐AP5 D4R activation failed to increase LTP.

D4R activation can suppress IPSCs by inhibiting the PKA‐protein phosphatase 1 pathway (Wang *et al*., 2002; Trantham‐Davidson *et al*., 2004). GABAergic inhibition opposes LTP induction (Grover & Yan, 1999; Ishizeki *et al*., 2008) and HFS potentiates IPSCs through a presynaptic mechanism that requires PKA activation (Shew *et al*., 2000). Because in area CA1 D4Rs are mainly expressed on interneurons (Romo‐Parra *et al*., 2005) and our experiments were performed in slices with normal inhibition, it is therefore likely that D4R activation reduces IPSCs and prevent HFS‐induced potentiation of IPSCs, and so facilitate LTP induction in CA1.

Why would modulation of CA1 LTP by D4R‐mediated control of inhibition be age‐dependent? VDCC‐dependent CA1 LTP is very sensitive to GABAergic inhibition (Grover & Yan, 1999), but NMDA‐dependent CA1 LTP is not (Debray *et al*., 1997; Grover & Yan, 1999). This may explain why D4R activation potentiates LTP more in slices from aged mice where LTP is likely to depend more on VDCC‐mediated Ca^2+^ influx.

### Synergistic potentiation of D4R‐mediated LTP enhancement by VDCC inhibition

In the presence of the VDCC blocker nifedipine, D4R activation caused a strong enhancement of LTP in slices from aged mice. The D4R‐induced increase in LTP magnitude in the presence of nifedipine was more than that of nifedipine alone plus that of D4R alone. In contrast, in the presence of the NMDAR blocker D‐AP5, D4R activation was unable to increase LTP and even prevented LTP induction in slices from aged mice. This indicates that the Ca^2+^ source is critical for the effect of D4R activation on LTP.

Increased Ca^2+^ influx through VDCCs during the HFS may suppress NMDAR‐mediated Ca^2+^ influx (Wang *et al*., 2003; Herwerth *et al*., 2012) in slices from aged rats. Whereas in adult neurons D4R activation inhibits NMDARs, in aged neurons, VDCC blockade may overturn the D4R mediated inhibition of NMDARs. If VDCC blockade in this situation actually disinhibits NMDARs, this can provide the HFS‐specific Ca^2+^ influx required for full expression of the D4R‐mediated enhancement of LTP. This hypothesis is supported by the observation that the cell‐permeable Ca^2+^ chelator BAPTA, which restores the impaired Ca^2+^ dynamics in hippocampal CA1 neurons of aged rats, improves synaptic plasticity (Tonkikh *et al*., 2006). The detailed mechanism regarding such a potentiation of LTP in aging mice is currently unclear and requires further study of the Ca^2+^ related signaling pathways involved. In addition, a role for potential changes in inhibition in this enhancement of LTP cannot be ruled out at this stage.

### Clinical relevance of the enhancement of LTP by D4R activation in aging

The enhancement of LTP by D4R activation indicates that the aging‐associated LTP impairment is reversible and suggests that D4R agonist may therefore restore cognitive function in memory‐impaired aged people. In line with this, individual differences in dopamine functions can be linked to cognitive performance in humans and serve as powerful mediators of age‐related decline in executive functioning, episodic memory, and perceptual speed (Backman *et al*., 2010). Furthermore, boosting dopaminergic neurotransmission in aged human subjects can indeed facilitate episodic memory (Chowdhury *et al*., 2013).

The synergistic effect of simultaneous D4R activation and VDCC blockade on facilitation of SC‐CA1 LTP suggests a potential therapeutic role for VDCC blockers in aging‐associated memory impairment. Interestingly, restoring Ca^2+^ dynamics in hippocampal CA1 neurons of aged rats by the VDCC blocker nimodipine or the cell‐permeable Ca^2+^ chelator BAPTA enhances spatial learning (Tonkikh *et al*., 2006).

The synergistic action of D4R activation and VDCC inhibition on CA1 LTP in slices from aged mice predicts that even more cognitive benefit can be achieved in memory‐impaired aged humans by a combination of D4R agonist and VDCC inhibitors.

## Experimental procedures

### Animal treatments and ethics

All experimental protocols (protocol number: 11401300017419) were approved by the Animal Experimentation Ethics Committee of Xinxiang Medical University, and all efforts were made to minimize animal suffering and reduce the number of animals used. All experiments were performed in accordance with the guidelines of the Animal Care and Use Committee of Xinxiang Medical University.

### Pharmacological agents and reagents

The high‐affinity, selective D4R agonist *N*‐(Methyl‐4‐(2‐cyanophenyl) piperaziny l – 3‐methylbenzamide maleate (PD168077), the highly selective D_4_ dopamine receptor antagonis 3‐(4‐piperazin‐1‐yl)‐methyl‐1*H*‐pyrrolo [2,3‐*b*] pyridine trihydrochloride (L745870), the dopamine D_1_‐like receptor agonist (±)‐6‐Chloro‐2,3,4,5‐tetrahydro‐1‐phenyl‐1*H*‐3‐benzazepine hydrobromide (SKF‐81297), the endogenous DR agonist 3,4‐Dihydroxyphenethylamine (dopamine), the NMDAR antagonist D‐(–)‐2‐amino‐5‐phosphonopentanoic acid (D‐AP5), the L‐type VDCC blocker 1,4‐Dihydro‐2,6‐dimethyl‐4‐(2‐nitrophenyl)‐3, 5‐pyridinedicarboxylic acid dimethyl ester (nifedpine), the D1R/D5R antagonist R(+)‐7‐Chloro‐8‐hydroxy‐3‐methyl‐1‐phenyl‐2,3,4,5‐tetrahydro‐1H‐3‐benzazepine hydrochloride (SCH23390), the D2R/D3R/D4R antagonist (S)‐3,5‐Dichloro‐N‐[(1‐ethyl‐2‐pyrrolidinyl)methyl]‐2,6‐dihydroxybenzamide hydrobromide (raclopride), and the selective and competitive AMPA receptor antagonist 2,3‐Dioxo‐6‐nitro‐1,2,3,4 –tetrahydrobenzo [*f*] quinoxaline‐7‐sulfonamide (NBQX) were purchased from Tocris Cookson Ltd (Bristol, UK). Stock solutions, at thousand times the final concentration, were made up in water, except for NBQX, which was dissolved in dimethylsulfoxide. Stock solutions were stored in individual aliquots at −20 °C. Final solutions were prepared freshly on the day of the experiment.

### Preparation of hippocampal slices

Mice (c57bl/6n) used in this experiment were sourced from the Institute of Medical Laboratory Animals, Chinese Academy of Medical Sciences and bred in our own animal facilities. Mice were kept in standard housing conditions: 4–5 mice per cage, with normal chow and water *ad libitum*, under a normal 6 am light, 6 pm dark cycle. Mice were taken from the ‘adult’ group (3‐ to 4‐month‐old males) or the ‘aged’ group (20‐ to 24‐month‐old males). Mice were anesthetized with chloral hydrate and perfused with chilled (0 °C) sucrose‐based cutting solution through the left ventricle until the limbs turned white. The brain was then rapidly removed and immersed in chilled sucrose‐based cutting solution containing (in mm): 225 sucrose; 3 KCl, 6 MgCl_2_, 1.25 NaH_2_PO_4_, 24 NaHCO_3_, 0.5 CaCl_2_, 10 glucose. Horizontal 350‐μm‐thick slices, containing the ventral hippocampus, were cut using a Leica VT1000S vibratome (Leica Microsystems UK, Milton Keynes, UK). Slices containing the ventral hippocampus were then transferred to an incubation chamber, where they remained submerged in oxygenated aCSF, which consisted of (in mm), 126 NaCl, 3 KCl, 1.25 NaH_2_PO_4_, 2 MgSO_4_, 24 NaHCO_3_, 2 CaCl_2_, and 10 glucose, pH 7.35–7.45 at room temperature until used for recording.

### Electrophysiological recordings

After at least 1 h of equilibration, slices were transferred to an interface‐type recording chamber where they were perfused with aCSF of 32 °C, at a rate of 4 mL min^−1^, with their surface exposed to warm, humidified carbogen (95% O_2_‐5% CO_2_). Field potentials were recorded from SR of CA1 (See Fig. 1A), using a glass pipette filled with aCSF (resistance was 2–3 MΩ). Recordings were band‐pass filtered online between 0.5 Hz and 2 kHz using an Axoprobe amplifier (Digitimer Ltd, Welwyn Garden City, UK) and a Neurolog system NL106 AC/DC amplifier (Digitimer Ltd, Welwyn Garden City, UK). The data were digitized at a sample rate of 10 kHz using a CED 1401 Plus ADC board (Digitimer Ltd). Electrical interference from the mains supply was online eliminated from extracellular recordings using HumBug noise eliminators (Digitimer Ltd). PSPs were evoked by orthodromic stimulation of the SC‐commissural fibers in CA1 SR (See Fig. 1A), using twisted 50 μm nickel/chromium wires. Pulses of 0.1 ms duration were delivered every 20 s. Pulses were given at varying stimulus intensity to establish a stimulus intensity–response relationship. The downward PSP slope was calculated between 10% and 50% of the maximum amplitude of the PSP (see Fig. 1B). The standard stimulus intensity was set at the intensity that evoked a PSP amplitude 50% of the maximum PSP amplitude. After a stable baseline (15–30 min) was established, LTP was induced by a single HFS train (100 Hz, 1 s at standard intensity), after which changes in the PSP slope were recorded for 60 min. To test the effect of drugs on LTP, drugs were applied to the aCSF 20–40 min before the HFS and were then present throughout the experiment.

### Western blotting

Protein was extracted from the hippocampus isolated from the brain of different age groups. Protein was denatured at 100 °C for 5 min and was separated by 10% sodium dodecyl sulfate–polyacrylamide gel electrophoresis (SDS‐PAGE). Protein was transferred onto PVDF membranes (Millipore, Boston, MA, USA) using a Mini PROTEAN Tetra Cell (Bio‐Rad, Hercules, CA, USA) following the manufacturer's instructions. Transferred membranes were blocked in 5% skim milk dissolved in Tris‐buffered saline pH 7.5/0.1% Tween‐20 (TBST) for 1.5 h at room temperature and then incubated at 4 °C overnight with one of the following primary antibodies: anti‐D1R (1:500, 50 kDa, Abcam) or anti‐D4R (1:500, 48 kDa, Abcam) and β‐actin (loading control, 1:2000, 42 kDa, Thermo Scientific). The membranes were washed three times with TBST and then incubated with appropriate conjugated hydrogen peroxidase HRP secondary antibody (1:2000, Abcam) for 1 h at room temperature. Proteins were visualized using the enhanced chemiluminescence (ECL) reagent (4A Biotech Co. Ltd, Shanghai, China) and quantified using ImageJ.

### Data analysis and statistics

Data were analyzed offline using software from Spike 2 (CED, Cambridge, UK). LTP was expressed as the PSP slope and normalized to the average PSP slope over the last 10 min before LTP induction.

All statistical tests were performed using SigmaStat software (SPSS Inc., California, USA). Data sets were tested for normality using the Shapiro–Wilk test, but no deviation from a normal distribution was detected and results are expressed as mean ± standard error of mean. Comparison of HFS‐induced plasticity was performed between groups using two‐way repeated‐measures ANOVA. This analysis was followed by a post hoc *t*‐test performed between groups. Differences between groups and changes within groups were considered statistically significant if *P* < 0.05.

## Funding

This study was supported by the National Natural Science Foundation of China (NSFC, grant numbers: 81771517; 81271422; 31070938).

## Author contributions

F.G. performed research, analyzed data, and wrote the study; J. Z. and Z.F. analyzed data; J.W., D.Z., and X.W. analyzed data and provided the technical supports; M.V. wrote the study; C.L. designed research, analyzed data, and wrote the study.

## Conflict of interest

None declared.
